# (*E*)-2-[4-(Trifluoro­meth­yl)benzyl­idene]-2,3-dihydro-1*H*-inden-1-one

**DOI:** 10.1107/S1600536812003157

**Published:** 2012-01-31

**Authors:** Ang Chee Wei, Mohamed Ashraf Ali, Tan Soo Choon, Ibrahim Abdul Razak, Suhana Arshad

**Affiliations:** a Institute for Research in Molecular Medicine, Universiti Sains Malaysia, Minden 11800, Penang, Malaysia; b School of Physics, Universiti Sains Malaysia, 11800 USM, Penang, Malaysia

## Abstract

In the title mol­ecule, C_17_H_11_F_3_O, the indan ring system and the trifluoro­methyl-substituted benzene ring are approximately individually planar and form a dihedral angle of 1.81 (5)° with each other. In the crystal, mol­ecules are linked by pairs of weak bifurcated (C—H)_2_⋯O hydrogen bonds to form centrosymmetric dimers, generating *R*_2_^1^(6) and *R*_2_^2^(10) ring motifs. These dimers are connected by further weak C—H⋯O hydrogen bonds into one-dimensional chains along the *b* axis. Weak C—H⋯π inter­actions are also present.

## Related literature

For the biological activity of chalcone compounds, see: Gurubasavaraja Swamy & Agasimundin (2008 ▶); Shibata (1994 ▶); Charris *et al.* (2007 ▶); Sharma *et al.* (2009 ▶). For related structures, see: Ali *et al.* (2011*a* ▶,*b* ▶,*c* ▶). For hydrogen-bond motifs, see: Bernstein *et al.* (1995 ▶). For the stability of the temperature controller used for data collection, see: Cosier & Glazer (1986 ▶).
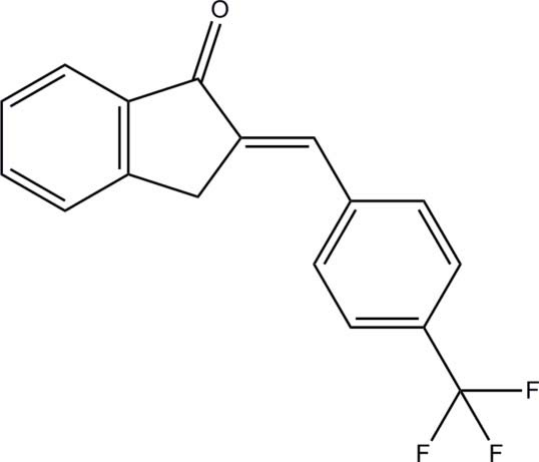


## Experimental

### 

#### Crystal data

C_17_H_11_F_3_O
*M* *_r_* = 288.26
Monoclinic, 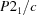

*a* = 15.6546 (13) Å
*b* = 6.2050 (6) Å
*c* = 14.6546 (13) Å
β = 113.774 (2)°
*V* = 1302.7 (2) Å^3^
*Z* = 4
Mo *K*α radiation
μ = 0.12 mm^−1^
*T* = 100 K
0.40 × 0.18 × 0.10 mm

#### Data collection

Bruker SMART APEXII CCD diffractometer
Absorption correction: multi-scan (*SADABS*; Bruker, 2009 ▶) *T*_min_ = 0.954, *T*_max_ = 0.988
10643 measured reflections
3804 independent reflections
3033 reflections with *I* > 2σ(*I*)
*R*_int_ = 0.023

#### Refinement

*R*[*F* ^2^ > 2σ(*F* ^2^)] = 0.046
*wR*(*F* ^2^) = 0.129
*S* = 1.06
3804 reflections
190 parameters
H-atom parameters constrained
Δρ_max_ = 0.55 e Å^−3^
Δρ_min_ = −0.32 e Å^−3^

### 

Data collection: *APEX2* (Bruker, 2009 ▶); cell refinement: *SAINT* (Bruker, 2009 ▶); data reduction: *SAINT*; program(s) used to solve structure: *SHELXTL* (Sheldrick, 2008 ▶); program(s) used to refine structure: *SHELXTL*; molecular graphics: *SHELXTL*; software used to prepare material for publication: *SHELXTL* and *PLATON* (Spek, 2009 ▶).

## Supplementary Material

Crystal structure: contains datablock(s) global, I. DOI: 10.1107/S1600536812003157/lh5408sup1.cif

Structure factors: contains datablock(s) I. DOI: 10.1107/S1600536812003157/lh5408Isup2.hkl

Supplementary material file. DOI: 10.1107/S1600536812003157/lh5408Isup3.cml

Additional supplementary materials:  crystallographic information; 3D view; checkCIF report

## Figures and Tables

**Table 1:** Hydrogen-bond geometry (Å, °) *Cg*1 and *Cg*2 are the centroids of the C2–C7 and C11–C16 rings, respectively.

*D*—H⋯*A*	*D*—H	H⋯*A*	*D*⋯*A*	*D*—H⋯*A*
C1—H1*B*⋯O1^i^	0.99	2.45	3.2713 (17)	140
C10—H10*A*⋯O1^ii^	0.95	2.54	3.3566 (17)	144
C12—H12*A*⋯O1^ii^	0.95	2.45	3.2765 (17)	146
C15—H15*A*⋯*Cg*1^iii^	0.95	2.78	3.5163 (14)	135
C3—H3*A*⋯*Cg*2^iii^	0.95	2.81	3.5035 (15)	130

Symmetry codes: (i) 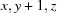; (ii) 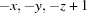; (iii) 
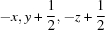
.

**Fig. 1.**
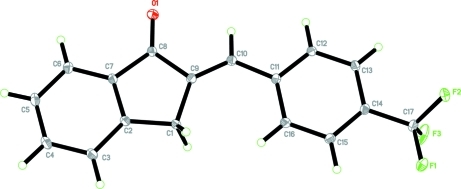
The molecular structure of the title compound, showing 30% probability displacement ellipsoids.

**Fig. 2.**
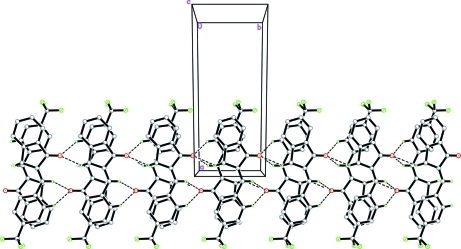
The crystal packing of the title compound. The H atoms not involved in the intermolecular interactions (dashed lines) have been omitted for clarity.

## supplementary crystallographic information

### Comment

Chalcones are an important group of natural products and many of these compounds possess various biological activities including antibacterial (Gurubasavaraja Swamy & Agasimundin, 2008), antitumor (Shibata, 1994), antimalarial (Charris *et al.*, 2007) and antitubercular (Sharma *et al.*, 2009). Indanones have been studied extensively as they are very useful intermediates for the synthesis of heterocyclic compounds. As part of our ongoing search to discover novel indanone related compounds (Ali *et al.*, 2011*a*,*b*) our group has synthesized the title compound as described below.

In the molecular structure (Fig 1), the 2,3-dihydro-1*H*-indene ring system (C1–C9) and the benzene ring (C11–C16) are approximately planar with a dihedral angle of 1.81 (5)° between them. The bond lengths and angles are within normal ranges and comparable to the related structure (Ali *et al.*, 2011*c*).

The crystal packing is shown in Fig. 2. The molecules are linked by intermolecular C1—H1B···O1^i^, C10—H10A···O1^ii^ and C12—H12A···O1^ii^ interactions (Table 1) to form dimers, generating *R*^1^_2_(6) and *R*^2^_2_(10) ring motifs (Bernstein *et al.*, 1995). Furthermore, these sets of ring motifs are connected into one-dimensional chains along the *b*-axis. In addition, the crystal structure is further stabilized by weak intermolecular C15—H15A···*Cg1*^iii^ and C3—H3A···*Cg2*^iii^ (Table 1) interactions (*Cg*1 and *Cg*2 are the centroids of C2–C7 and C11–C16 rings, respectively).

### Experimental

A mixture of 2,3-dihydro-1*H*-indene-1-one (0.001 mol) and 4-(trifluoromethyl)benzaldehyde (0.001 mol) were dissolved in ethanolic sodium hydroxide solution (15 ml) and the mixture was stirred for 5 h. After completion of the reaction as evident from TLC, the mixture was poured into crushed ice then neutralized with concentrated HCl. The precipitated solid was filtered, washed with water and recrystallized from ethanol to reveal the title compound as yellow crystals.

### Refinement

All H atoms were positioned geometrically [C—H = 0.95 and 0.99 Å] and refined using a riding model with *U*_iso_(H) = 1.2 *U*_eq_(C). Two outliers were omitted for the final refinement, 3 4 4 and 3 4 3.

### Crystal data

**Table:**

C_17_H_11_F_3_O	*F*(000) = 592
*M**_r_* = 288.26	*D*_x_ = 1.470 Mg m^−^^3^
Monoclinic, *P*2_1_/*c*	Mo *K*α radiation, λ = 0.71073 Å
Hall symbol: -P 2ybc	Cell parameters from 3626 reflections
*a* = 15.6546 (13) Å	θ = 2.8–30.1°
*b* = 6.2050 (6) Å	µ = 0.12 mm^−^^1^
*c* = 14.6546 (13) Å	*T* = 100 K
β = 113.774 (2)°	Plate, colourless
*V* = 1302.7 (2) Å^3^	0.40 × 0.18 × 0.10 mm
*Z* = 4	

### Data collection

**Table:**

Bruker SMART APEXII CCD diffractometer	3804 independent reflections
Radiation source: fine-focus sealed tube	3033 reflections with *I* > 2σ(*I*)
graphite	*R*_int_ = 0.023
φ and ω scans	θ_max_ = 30.1°, θ_min_ = 2.8°
Absorption correction: multi-scan (*SADABS*; Bruker, 2009)	*h* = −21→22
*T*_min_ = 0.954, *T*_max_ = 0.988	*k* = −8→8
10643 measured reflections	*l* = −20→20

### Refinement

**Table:**

Refinement on *F*^2^	Primary atom site location: structure-invariant direct methods
Least-squares matrix: full	Secondary atom site location: difference Fourier map
*R*[*F*^2^ > 2σ(*F*^2^)] = 0.046	Hydrogen site location: inferred from neighbouring sites
*wR*(*F*^2^) = 0.129	H-atom parameters constrained
*S* = 1.06	*w* = 1/[σ^2^(*F*_o_^2^) + (0.0567*P*)^2^ + 0.6847*P*] where *P* = (*F*_o_^2^ + 2*F*_c_^2^)/3
3804 reflections	(Δ/σ)_max_ < 0.001
190 parameters	Δρ_max_ = 0.55 e Å^−^^3^
0 restraints	Δρ_min_ = −0.32 e Å^−^^3^

### Special details

**Table:**

Experimental. The crystal was placed in the cold stream of an Oxford Cryosystems Cobra open-flow nitrogen cryostat (Cosier & Glazer, 1986) operating at 100.0 (1) K.
Geometry. All e.s.d.'s (except the e.s.d. in the dihedral angle between two l.s. planes) are estimated using the full covariance matrix. The cell e.s.d.'s are taken into account individually in the estimation of e.s.d.'s in distances, angles and torsion angles; correlations between e.s.d.'s in cell parameters are only used when they are defined by crystal symmetry. An approximate (isotropic) treatment of cell e.s.d.'s is used for estimating e.s.d.'s involving l.s. planes.
Refinement. Refinement of *F*^2^ against ALL reflections. The weighted *R*-factor *wR* and goodness of fit *S* are based on *F*^2^, conventional *R*-factors *R* are based on *F*, with *F* set to zero for negative *F*^2^. The threshold expression of *F*^2^ > σ(*F*^2^) is used only for calculating *R*-factors(gt) *etc*. and is not relevant to the choice of reflections for refinement. *R*-factors based on *F*^2^ are statistically about twice as large as those based on *F*, and *R*-factors based on ALL data will be even larger.

### Fractional atomic coordinates and isotropic or equivalent isotropic displacement parameters (Å^2^)

**Table:**

	*x*	*y*	*z*	*U*_iso_*/*U*_eq_	
F1	−0.41885 (6)	0.88298 (16)	0.17877 (8)	0.0335 (2)	
F2	−0.47050 (6)	0.62598 (19)	0.23994 (8)	0.0392 (3)	
F3	−0.45949 (7)	0.5856 (2)	0.09945 (8)	0.0450 (3)	
O1	0.11757 (7)	0.05902 (17)	0.48959 (8)	0.0225 (2)	
C1	0.07259 (8)	0.5913 (2)	0.37708 (10)	0.0170 (3)	
H1A	0.0478	0.6137	0.3041	0.020*	
H1B	0.0544	0.7149	0.4081	0.020*	
C2	0.17752 (8)	0.5624 (2)	0.42083 (9)	0.0158 (2)	
C3	0.24539 (9)	0.7074 (2)	0.42066 (10)	0.0192 (3)	
H3A	0.2285	0.8445	0.3897	0.023*	
C4	0.33894 (9)	0.6456 (3)	0.46729 (10)	0.0221 (3)	
H4A	0.3861	0.7427	0.4680	0.026*	
C5	0.36474 (9)	0.4440 (3)	0.51297 (10)	0.0215 (3)	
H5A	0.4289	0.4062	0.5440	0.026*	
C6	0.29734 (9)	0.2987 (2)	0.51332 (9)	0.0187 (3)	
H6A	0.3141	0.1614	0.5441	0.022*	
C7	0.20371 (8)	0.3623 (2)	0.46647 (9)	0.0154 (2)	
C8	0.12026 (8)	0.2400 (2)	0.45815 (9)	0.0166 (3)	
C9	0.03802 (8)	0.3802 (2)	0.40223 (9)	0.0163 (2)	
C10	−0.04785 (8)	0.3083 (2)	0.38672 (9)	0.0167 (2)	
H10A	−0.0496	0.1677	0.4116	0.020*	
C11	−0.13924 (8)	0.4128 (2)	0.33745 (9)	0.0164 (2)	
C12	−0.21766 (8)	0.2952 (2)	0.33344 (9)	0.0159 (2)	
H12A	−0.2091	0.1570	0.3637	0.019*	
C13	−0.30716 (8)	0.3780 (2)	0.28612 (9)	0.0173 (3)	
H13A	−0.3595	0.2959	0.2829	0.021*	
C14	−0.31963 (8)	0.5819 (2)	0.24335 (9)	0.0166 (3)	
C15	−0.24327 (9)	0.7038 (2)	0.24696 (9)	0.0175 (3)	
H15A	−0.2523	0.8430	0.2176	0.021*	
C16	−0.15373 (9)	0.6189 (2)	0.29410 (10)	0.0182 (3)	
H16A	−0.1016	0.7016	0.2970	0.022*	
C17	−0.41652 (9)	0.6688 (3)	0.19105 (10)	0.0219 (3)	

### Atomic displacement parameters (Å^2^)

**Table:**

	*U*^11^	*U*^22^	*U*^33^	*U*^12^	*U*^13^	*U*^23^
F1	0.0274 (4)	0.0258 (5)	0.0519 (6)	0.0129 (4)	0.0208 (4)	0.0134 (5)
F2	0.0209 (4)	0.0532 (7)	0.0507 (6)	0.0122 (4)	0.0218 (4)	0.0260 (5)
F3	0.0284 (5)	0.0563 (8)	0.0316 (5)	0.0171 (5)	−0.0074 (4)	−0.0106 (5)
O1	0.0210 (4)	0.0179 (5)	0.0275 (5)	−0.0019 (4)	0.0086 (4)	0.0045 (4)
C1	0.0148 (5)	0.0169 (6)	0.0181 (5)	−0.0023 (5)	0.0055 (4)	0.0026 (5)
C2	0.0156 (5)	0.0179 (6)	0.0143 (5)	−0.0030 (5)	0.0065 (4)	−0.0011 (5)
C3	0.0192 (6)	0.0195 (7)	0.0196 (6)	−0.0044 (5)	0.0086 (5)	−0.0004 (5)
C4	0.0177 (6)	0.0274 (8)	0.0227 (6)	−0.0077 (5)	0.0097 (5)	−0.0034 (6)
C5	0.0143 (5)	0.0296 (8)	0.0197 (6)	−0.0025 (5)	0.0061 (5)	−0.0029 (6)
C6	0.0161 (5)	0.0218 (7)	0.0175 (5)	−0.0004 (5)	0.0059 (4)	−0.0018 (5)
C7	0.0140 (5)	0.0178 (6)	0.0145 (5)	−0.0026 (5)	0.0059 (4)	−0.0015 (5)
C8	0.0145 (5)	0.0179 (6)	0.0162 (5)	−0.0016 (5)	0.0050 (4)	−0.0003 (5)
C9	0.0162 (5)	0.0154 (6)	0.0165 (5)	−0.0003 (5)	0.0059 (4)	−0.0002 (5)
C10	0.0159 (5)	0.0157 (6)	0.0176 (5)	0.0001 (5)	0.0059 (4)	−0.0002 (5)
C11	0.0141 (5)	0.0178 (6)	0.0155 (5)	−0.0006 (5)	0.0042 (4)	0.0008 (5)
C12	0.0146 (5)	0.0157 (6)	0.0164 (5)	−0.0010 (5)	0.0053 (4)	0.0008 (5)
C13	0.0141 (5)	0.0194 (6)	0.0186 (6)	−0.0013 (5)	0.0069 (4)	0.0002 (5)
C14	0.0149 (5)	0.0191 (6)	0.0161 (5)	0.0032 (5)	0.0065 (4)	0.0002 (5)
C15	0.0185 (6)	0.0163 (6)	0.0176 (5)	0.0019 (5)	0.0071 (4)	0.0021 (5)
C16	0.0163 (5)	0.0180 (6)	0.0190 (6)	−0.0020 (5)	0.0057 (4)	0.0023 (5)
C17	0.0174 (6)	0.0252 (7)	0.0236 (6)	0.0059 (5)	0.0088 (5)	0.0054 (6)

### Geometric parameters (Å, °)

**Table:**

F1—C17	1.3395 (18)	C6—H6A	0.9500
F2—C17	1.3365 (16)	C7—C8	1.4727 (17)
F3—C17	1.3391 (18)	C8—C9	1.4951 (18)
O1—C8	1.2211 (17)	C9—C10	1.3460 (17)
C1—C2	1.5141 (17)	C10—C11	1.4683 (17)
C1—C9	1.5180 (19)	C10—H10A	0.9500
C1—H1A	0.9900	C11—C16	1.4049 (19)
C1—H1B	0.9900	C11—C12	1.4090 (17)
C2—C7	1.3912 (19)	C12—C13	1.3871 (17)
C2—C3	1.3932 (18)	C12—H12A	0.9500
C3—C4	1.3971 (19)	C13—C14	1.3907 (19)
C3—H3A	0.9500	C13—H13A	0.9500
C4—C5	1.399 (2)	C14—C15	1.3974 (18)
C4—H4A	0.9500	C14—C17	1.4972 (17)
C5—C6	1.3895 (19)	C15—C16	1.3926 (18)
C5—H5A	0.9500	C15—H15A	0.9500
C6—C7	1.4016 (17)	C16—H16A	0.9500
			
C2—C1—C9	103.13 (11)	C8—C9—C1	108.82 (10)
C2—C1—H1A	111.1	C9—C10—C11	130.19 (13)
C9—C1—H1A	111.1	C9—C10—H10A	114.9
C2—C1—H1B	111.1	C11—C10—H10A	114.9
C9—C1—H1B	111.1	C16—C11—C12	118.25 (11)
H1A—C1—H1B	109.1	C16—C11—C10	124.90 (11)
C7—C2—C3	120.00 (12)	C12—C11—C10	116.85 (12)
C7—C2—C1	111.62 (11)	C13—C12—C11	121.11 (12)
C3—C2—C1	128.37 (12)	C13—C12—H12A	119.4
C2—C3—C4	118.14 (13)	C11—C12—H12A	119.4
C2—C3—H3A	120.9	C12—C13—C14	119.48 (12)
C4—C3—H3A	120.9	C12—C13—H13A	120.3
C3—C4—C5	121.53 (13)	C14—C13—H13A	120.3
C3—C4—H4A	119.2	C13—C14—C15	120.86 (12)
C5—C4—H4A	119.2	C13—C14—C17	119.19 (12)
C6—C5—C4	120.58 (12)	C15—C14—C17	119.94 (12)
C6—C5—H5A	119.7	C16—C15—C14	119.25 (13)
C4—C5—H5A	119.7	C16—C15—H15A	120.4
C5—C6—C7	117.46 (13)	C14—C15—H15A	120.4
C5—C6—H6A	121.3	C15—C16—C11	121.03 (12)
C7—C6—H6A	121.3	C15—C16—H16A	119.5
C2—C7—C6	122.29 (12)	C11—C16—H16A	119.5
C2—C7—C8	109.90 (11)	F2—C17—F3	106.87 (13)
C6—C7—C8	127.81 (13)	F2—C17—F1	106.30 (12)
O1—C8—C7	127.37 (12)	F3—C17—F1	105.80 (12)
O1—C8—C9	126.09 (12)	F2—C17—C14	112.52 (11)
C7—C8—C9	106.53 (11)	F3—C17—C14	111.72 (12)
C10—C9—C8	118.70 (12)	F1—C17—C14	113.15 (12)
C10—C9—C1	132.43 (12)		
			
C9—C1—C2—C7	−0.08 (14)	C2—C1—C9—C8	0.30 (13)
C9—C1—C2—C3	−179.37 (13)	C8—C9—C10—C11	178.11 (12)
C7—C2—C3—C4	−0.10 (19)	C1—C9—C10—C11	1.1 (2)
C1—C2—C3—C4	179.14 (13)	C9—C10—C11—C16	0.8 (2)
C2—C3—C4—C5	0.2 (2)	C9—C10—C11—C12	−179.53 (13)
C3—C4—C5—C6	−0.1 (2)	C16—C11—C12—C13	1.41 (19)
C4—C5—C6—C7	0.02 (19)	C10—C11—C12—C13	−178.26 (12)
C3—C2—C7—C6	0.01 (19)	C11—C12—C13—C14	−1.11 (19)
C1—C2—C7—C6	−179.36 (12)	C12—C13—C14—C15	0.38 (19)
C3—C2—C7—C8	179.18 (11)	C12—C13—C14—C17	179.09 (12)
C1—C2—C7—C8	−0.18 (15)	C13—C14—C15—C16	0.01 (19)
C5—C6—C7—C2	0.03 (19)	C17—C14—C15—C16	−178.69 (12)
C5—C6—C7—C8	−178.99 (12)	C14—C15—C16—C11	0.3 (2)
C2—C7—C8—O1	179.80 (13)	C12—C11—C16—C15	−1.01 (19)
C6—C7—C8—O1	−1.1 (2)	C10—C11—C16—C15	178.63 (12)
C2—C7—C8—C9	0.36 (14)	C13—C14—C17—F2	41.38 (18)
C6—C7—C8—C9	179.48 (12)	C15—C14—C17—F2	−139.90 (14)
O1—C8—C9—C10	2.5 (2)	C13—C14—C17—F3	−78.84 (16)
C7—C8—C9—C10	−178.09 (11)	C15—C14—C17—F3	99.88 (16)
O1—C8—C9—C1	−179.86 (13)	C13—C14—C17—F1	161.88 (12)
C7—C8—C9—C1	−0.41 (14)	C15—C14—C17—F1	−19.40 (18)
C2—C1—C9—C10	177.55 (14)		

### Hydrogen-bond geometry (Å, °)

**Table:**

Cg1 and Cg2 are the centroids of the C2–C7 and C11–C16 rings, respectively.

**Table:**

*D*—H···*A*	*D*—H	H···*A*	*D*···*A*	*D*—H···*A*
C1—H1B···O1^i^	0.99	2.45	3.2713 (17)	140.
C10—H10A···O1^ii^	0.95	2.54	3.3566 (17)	144.
C12—H12A···O1^ii^	0.95	2.45	3.2765 (17)	146.
C15—H15A···Cg1^iii^	0.95	2.78	3.5163 (14)	135.
C3—H3A···Cg2^iii^	0.95	2.81	3.5035 (15)	130.

Symmetry codes: (i) *x*, *y*+1, *z*; (ii) −*x*, −*y*, −*z*+1; (iii) −*x*, *y*+1/2, −*z*+1/2.
